# Beyond Dyadic Coupling: The Method of Multivariate Surrogate Synchrony (mv-SUSY)

**DOI:** 10.3390/e23111385

**Published:** 2021-10-22

**Authors:** Deborah Meier, Wolfgang Tschacher

**Affiliations:** University Hospital of Psychiatry and Psychotherapy, University of Bern, 3000 Bern 60, Switzerland; wolfgang.tschacher@upd.unibe.ch

**Keywords:** surrogate synchrony, multivariate analysis, simulation, movement synchrony

## Abstract

Measuring interpersonal synchrony is a promising approach to assess the complexity of social interaction, which however has been mostly limited to dyads. In this study, we introduce multivariate Surrogate Synchrony (mv-SUSY) to extend the current set of computational methods. Methods: mv-SUSY was applied to eight datasets consisting of 10 time series each, all with n = 9600 observations. Datasets 1 to 5 consist of simulated time series with the following characteristics: white noise (dataset 1), non-stationarity with linear time trends (dataset 2), autocorrelation (dataset 3), oscillation (dataset 4), and multivariate correlation (dataset 5). Datasets 6 to 8 comprise empirical multivariate movement data of two individuals (datasets 6 and 7) and between members of a group discussion (dataset 8.) Results: As hypothesized, findings of mv-SUSY revealed absence of synchrony in datasets 1 to 4 and presence of synchrony in dataset 5. In the empirical datasets, mv-SUSY indicated significant movement synchrony. These results were predominantly replicated by two well-established dyadic synchrony approaches, Surrogate Synchrony (SUSY) and Surrogate Concordance (SUCO). Conclusions: The study applied and evaluated a novel synchrony approach, mv-SUSY. We demonstrated the feasibility and validity of estimating multivariate nonverbal synchrony within and between individuals by mv-SUSY.

## 1. Introduction

Interpersonal synchrony has become a growing field of empirical research in social psychology and psychotherapy. Synchrony, composed of the Greek words syn (together) and chronos (time), denotes the coordination of variables that each represent the temporal evolution of the momentary state of a system. The bulk of applications has focused on the synchrony of two individuals A and B in social interaction. If these individuals act “together in time”, it is expected that their behavior is coupled and mutually entrained to a degree exceeding random correlations: A and B are then said to be in synchrony. The basic level of synchrony occurs at a phasic time scale of a few seconds; this is the time scale of the “Now”, the experienced present moment, during which present-time consciousness of the “here-and-now” arises [1]. This “Now” has a temporal duration, which provides a reason for including lagged correlations. At this time scale, synchrony can be detected in A and B’s body movement [2,3], neural activity [4], or physiological activations [5,6]. Accordingly, to quantify synchrony the variable of interest is observed continuously over time and the corresponding time series must be sampled at high temporal resolution. Temporal delays (lags) of the time series may be considered to account for the durational aspects of the “Now” as well as the psychological reality of response times in social interaction.

Various fields of psychological research have used synchrony measures. Since each field has its own terminology, several synonyms for the phenomenon of synchrony are used, such as attunement, interpersonal physiology, concordance, coupling or entrainment. The largest number of applications is in clinical psychology, where the coordination of therapist and patient in a therapy session is a phenomenon of major interest. Many studies were conducted addressing nonverbal measures, as participants’ body movements can be economically sampled by Motion Energy Analysis [7,8], a software package for automated motion capture from video recordings. Generally, studies have found that levels of movement synchrony significantly exceeded random control conditions [8,9,10]. It was often reported that synchrony had pro-social effects [11], predicted better outcomes, such as reduced interpersonal problems of patients, and was associated with the quality of the therapeutic alliance. Prosodic aspects of speech, especially the attunement of pitch and loudness of verbal utterances, can also be monitored in a non-invasive way in psychotherapy sessions [12]. Increasingly, synchrony is studied also on the basis of physiological variables of two participants. For an extensive literature review see Palumbo et al. [13]. This field has seen early forerunners in DiMascio’s group, who focused on interpersonal synchrony of heart rate in psychiatric interviews [14]. Marci & Orr [15] found concordance of electrodermal activity in clinical interviews, and this approach was also chosen in a setting of naturalistic couple therapies with two therapists [16]. Tschacher and Meier [6] investigated the synchrony of heart rate, heart-rate variability and respiration in psychotherapy sessions.

Fields of research outside clinical applications concerned social psychology [17], such as instructing dyads to have topical conversations [3], or cooperation tasks [18]. Synchrony of team performance has been studied, for instance, by Guastello, Mirabiti, and Peressini [19]. Findings have been that synchrony, also labeled mimicry, arises unintentionally between interacting participants, and increases the smoothness of social interactions, mutual liking, and positive affect. Furthermore, researchers in the field of synchrony have focused on close relationships such as romantic couples [20,21], co-parenting spouses [22], and mother-infant dyads [23]. Some applications have addressed music psychology and analyzed movement synchrony of musicians in concerts [24], physiological synchrony in concert audiences [25] and audiences of dance performance [26].

Synchrony was analyzed using a variety of computational methods, which generally consider the synchrony between two univariate time series. There are three clusters of such methods: Correlational methods (time-based), Fourier analysis (frequency-based) and cross-recurrence quantification (nonparametric). The time-based and frequency-based approaches are mathematically related because Fourier spectra in the limit can be transformed to correlational terms. Frequency-based methods have made use of wavelet transforms of the dual time series, and cross-wavelet coherence was then used as a synchrony measure, e.g., by Fujiwara and Daibo [2]. Cross-recurrence plots illustrate the dyadic time series A, B in the two-dimensional coordinate system, where the axes are given by ‘A plus/minus a time lag’ and ‘B plus/minus a time lag’. Regular behavior of A and B such as corresponding oscillations then generates regular patterns in the cross-recurrence plot that can be quantified to provide a measure of synchrony [27]. Published research on the quantification of multivariate synchrony is very limited. Multivariate methods include phase synchronization based on Kuramoto order [28,29], multidimensional recurrence quantification analysis [30], and matrix calculations to determine the “driver” and “empath” of a group of interacting persons, and based on this, to estimate a synchronization coefficient [31].

Correlational methods underlie a majority of applications in the synchrony literature; they are either based on the cross-correlation function (CCF) of A and B, which denotes the correlation between A and B depending on their lags, or on the correlation of A and B’s local slopes, which defines the concordance measure [15]. An elaborated variant of the cross-correlational approach is computing the CCF segment-wise to account for non-stationarity due to overarching trends or seasonality of the time series, which may lead to inflated correlations. This principle forms the core of windowed cross-correlation (WCC) analysis, where “segment-wise” is referred to as “windowed”. WCC analysis requires the specification of multiple parameters: segment size, segment increment, maximum lag, and lag increment. For statistical and theoretical considerations in WCC analysis see for example Behrens et al. [32]. Accordingly, differences in results between computational approaches often arise from different choices of one or more of these parameters. In this respect, surrogate synchrony (SUSY) has fixed segment increment and lag increment, which keeps the dependency on parameter settings low. In SUSY the time series is cut into segments (e.g., of 30 s duration) and all cross-correlations of this segment’s CCF within a certain range of lags (e.g., lags of up to ±3 s) are computed. Aggregation is done by transforming cross-correlations to (absolute) values of Fisher’s *Z* and computing the mean *Z* for each lag across segments. Aggregation is repeated across all lags, thus delivering a signature of synchrony by the mean *Z* (${\bar{Z}}_{\mathrm{noabs}}$) or by the mean of all absolute values of *Z* (${\bar{Z}}_{\mathrm{abs}}$). SUSY does not use overlapping segments (hence, segment increment = segment size) or lag increment (hence, lag increment is determined by the sampling rate of the time series).

Well-established approaches share the common characteristic of keeping parameter settings constant throughout the analysis. Thus, these methods do not interfere with the equidistance between time points. A very recent approach, dynamic time warping, is based on varying lags which allows stretching the time series to accordingly estimate synchrony [33]. Since parameter settings have the potential to influence the results to a considerable degree, researchers in the field of synchrony aimed to define universal guidelines [34].

Surrogate analysis is an appropriate tool to establish the significance of aggregated cross-correlations, which indicate the measured ‘real’ synchrony, by comparing this synchrony to a control condition, called surrogate synchrony. Measured synchrony may be due to random fluctuations, and surrogate controls help to determine the level of randomness. Measured synchrony may even be inflated because of trends and/or autocorrelation in the time series. For example, we would expect to estimate a certain level of synchrony in the respiration of individuals, even though they did not even interact. This would be simply due to the periodic rhythm of inhaling and exhaling. Furthermore, elevated synchrony levels may derive from coordinated dynamics in human interaction (e.g., linguistic turn-taking) and therefore merely reflect patterns of shared task context, whereas we are interested in the unique levels of synchrony above what can be expected due to that particular task. Segment shuffling tests the null hypothesis that there is no synchrony difference between the measured time series and the sample of randomly shuffled time series, where the temporal intercorrelations are lost. Assuming the null hypothesis is rejected, temporal intercorrelations between the measured time series exist above chance level. Surrogate analysis must be adapted to the respective null hypothesis [35]. Accordingly, surrogate data may be generated by various methods such as data shuffling, segment shuffling, data sliding, or participant shuffling [36].

With the present study, we aim to extend the synchrony methodology to multivariate time series and thus go beyond the predominant analysis of dyadic synchronies toward more complex datasets. To do this, we will propose different methods of multivariate surrogate synchrony (mv-SUSY), and all methods use segment shuffling for surrogate analysis. The extension to multivariate time series was motivated by the need for synchrony measures when more than just two variables are considered coupled. In interpersonal contexts, we may be interested in analyzing the synchrony in groups of people rather than dyads only; in the single person, we may be interested in the joint coupling of several physiological variables at the same time, or in the coordinated movement of several body parts of the same person. To demonstrate its validity, mv-SUSY must accurately indicate the existence or non-existence of the synchrony phenomenon it is supposed to capture.

The goals of the validity tests conducted in the present study were threefold: First, we aimed to establish the validity of mv-SUSY in simulated data. We hypothesized (H1a) to demonstrate the absence of multivariate synchrony in stationary random time series (dataset 1), non-stationary random time series with linear trends (dataset 2), autocorrelated time series (dataset 3), and time series of regular oscillations (dataset 4). We further hypothesized (H1b) that the presence of synchrony would be detected and confirmed in mutually correlated time series (dataset 5). Second, we extended the application of mv-SUSY to examples of empirical movement data. We hypothesized (H2) to demonstrate multivariate synchrony in movement time series captured using Kinect (datasets 6 and 7) and motion energy analysis (dataset 8). Third, well-established methods for dyadic synchrony (Surrogate Synchrony; SUSY, Surrogate Concordance; SUCO) were applied as a control methodology. We hypothesized (H3) that the repeated application of dyadic SUSY and SUCO would validate the mv-SUSY findings of the first and second goals.

## 2. Materials and Methods

### 2.1. Simulated Multivariate Datasets

To illustrate the estimation of multivariate synchrony, we generated five datasets that simulate different types of temporal behavior. Each dataset comprises 10 time series, y_m_ (1 ≤ m ≤ 10), each consisting of 9600 observations y_m*t*_ (1 ≤ m ≤ 10; 1 ≤ *t* ≤ 9600).

Dataset 1 was constructed to represent stationary random time series, i.e., white noise. The time series y_m_ consist of normally distributed random values, and time series are independent of each other. The distributions of values have mean = 0 and standard deviation = 1.

Dataset 1: y_m*t*_~*N*(0,1) (1)

The next three datasets simulate characteristics and non-stationarities frequently found in empirical time series, such as stable time trends, autocorrelated evolutions, and oscillatory behavior. These characteristics can lead to increased levels of detected synchrony, which should be identified as spurious by the respective surrogate analyses leading to support of null hypotheses.

Dataset 2 was generated to express white noise data with varying but stable trends (positive and negative), thus non-stationary time series. We simulated 9600 observations as a linear function of time. For each time series in dataset 2 the respective intercepts, β_0_, and slope coefficients, β_1_, were sampled individually, where β_0_~*N*(0,25) and β_1_~*N*(0,0.000001). We added some noise, ε*_t_*_,_ which was sampled from a distribution with mean = 0 and standard deviation = 1.

Dataset 2: y_m*t*_ = β_0_ + β_1*t*_ + ε*_t_* (2)

Dataset 3 comprises autocorrelated time series with high lag 1 correlations between observations (so-called AR(1) processes). Again, we added an error term, where ε*_t_*~*N*(0,1).

Dataset 3: y_m*t*_ = 0.9 × y*_t_*_−1_ + ε*_t_* (3)

Dataset 4 consists of oscillatory time series produced by sine functions of varying frequencies and vertical shifts. For each time series in dataset 4, periods given by 2π/b were individually sampled with b~*N*(0.4,0.0064) and vertically shifted with d~*N*(0,1).

Dataset 4: y_m*t*_ = sin(b × *t*) + d (4)

Dataset 5: Multivariate correlations among time series in this dataset were realized by creating a white noise baseline time series, y_1_, for which we assumed y_1_~*N*(0,1). Based on y_1_, nine dependent time series, y_2_ to y_10_, were generated by repeatedly adding a certain degree of noise, where ε*_t_*~*N*(0,0.25). The intercorrelated times series of this dataset was expected to entail rejection of null hypotheses.

Dataset 5: y*_t_* = y_1_ + ε*_t_* (5)

**Figure 1:**
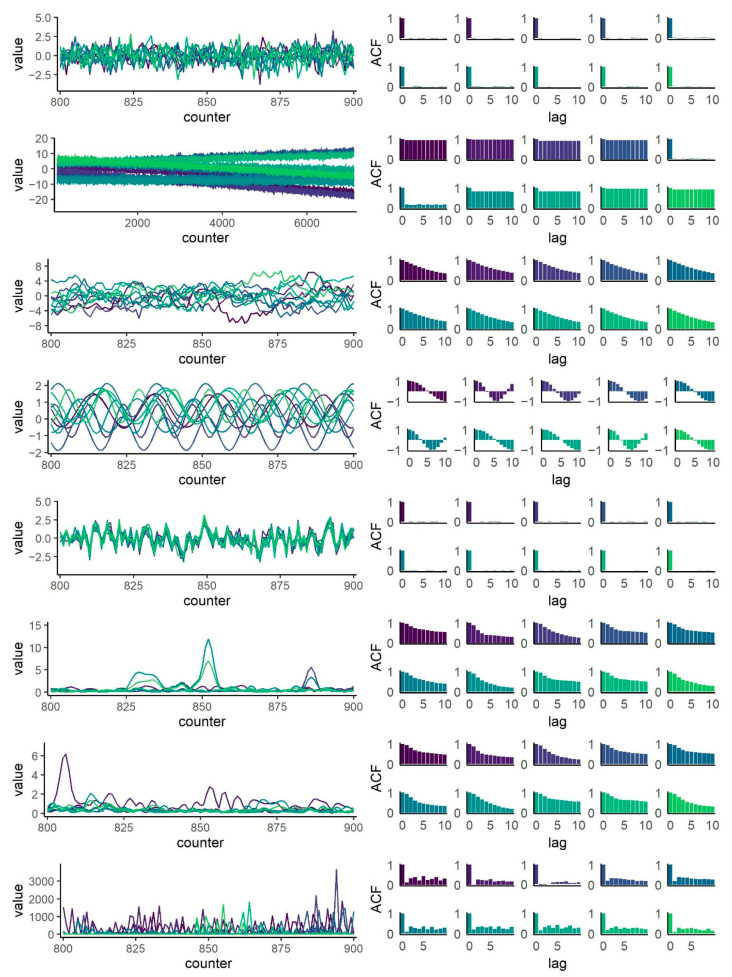
Multivariate datasets, each consisting of m = 10 time series (left panels, characteristic cutout plots) and autocorrelation functions (ACF, right panels). From top to bottom: simulated data (datasets 1 to 5), empirical data (datasets 6 to 8).

Visualizations of these datasets are given in Figure 1. The left panels correspond to the time series plots. The first five rows are time series plots of short sections (100 observations) of the simulated time series. The time series plot of dataset 2 shows 7000 observations to illustrate the long-range trends. The right panels of Figure 1 provide the ten autocorrelation functions (ACF) of each dataset up to lag 10. Autocorrelations indicate the different patterns of temporal dependence between single observations of the time series. Across datasets, time series were not autocorrelated (datasets 1 and 5), had stable autocorrelations (dataset 2). Dataset 3 showed the slowly decaying autocorrelation functions of autoregressive processes, dataset 4 the regular cyclical autocorrelation patterns of periodic data. Furthermore, the mean intercorrelations *r_m_* between the respective ten time series of each dataset demonstrated varying degrees of relationship within these samples: *r_m_*= 0.002 (dataset 1), *r_m_* = 0.12 (dataset 2), *r_m_* = 0.002 (dataset 3), *r_m_* = −0.00009 (dataset 4), and *r_m_* = 0.82 (dataset 5). Higher values for mean absolute intercorrelations indicated positive and negative pairwise correlations (dataset 1: *r_m_* = 0.01, dataset 2: *r_m_* = 0.65, dataset 3: *r_m_* = 0.03, dataset 4: *r_m_* = 0.002, dataset 5: *r_m_* = 0.82).

### 2.2. Empirical Multivariate Datasets

Datasets 6 to 8 represent empirical movement data. The Kinect datasets 6 and 7 originated from an experimental study, in which two participants were instructed to have a conversation exclusively with nonverbal expressions and dance movements in order “to try to get to know each other without words” [37]. Two previously unacquainted adults were allocated two non-overlapping areas marked on the floor, in which they could move freely without touching. All movements during the 5:20 minutes of this “body conversation task” were motion-captured using Kinect cameras, and time series consisted of 10 limb positions per person (head, chest, both upper arms, both lower arms, both upper legs, and both lower legs) at a sampling rate of 30 Hz.

The Zoom video data (dataset 8) consists of movement data recorded during an online group discussion among 10 individuals, whose body movements were captured by the automatized method MEA. MEA was based on pixel changes in assigned regions of interest of the Zoom video recording. The regions chosen were the panels that showed the respective head and upper body of the participants of the group discussion. Thus, the time series (sampling rate: 25 Hz) represent nonverbal movements of head, face, and upper body. The scholarly discussion was on the topic of “Embodiment, Physical Distancing and Treatment” (Virtual Structured Discussion, organized by the special interest group “Complexity in Psychotherapy” of the Society for Psychotherapy Research (SPR), 26 June 2020). All subjects agreed to the video recording. We selected the especially engaging final section of the Zoom discussion for further analysis. The section had a duration of 6 min 24 s (i.e., 9600 data points in the time series).

Visualizations of the empirical datasets are provided in Figure 1 (rows 6 to 8). On the left panels, there are time series plots of short sections (100 observations) of the empirical datasets. The right panels of Figure 1 provide the autocorrelation functions up to lag 10. Autocorrelations functions of the Kinect data (rows 6 and 7) resemble those of AR(1) autoregressive processes, whereas the Zoom dataset (row 8) had a pattern of smaller autocorrelations. The mean intercorrelations between the time series of each dataset were *r_m_* = 0.47 (dataset 6), *r_m_* = 0.48 (dataset 7), and *r_m_* = 0.01 (dataset 8). Negative correlations were only found in dataset 8, where the mean absolute intercorrelations *r_m_* = 0.02.

### 2.3. Dyadic Synchrony Computation

Surrogate Synchrony (SUSY, cf. www.embodiment.ch, accessed on 11 October 2021) estimates dyadic synchrony defined as cross-correlations between two time series A and B. The core procedure lies in the control of real synchrony by surrogate synchrony. Therefore, time series A and B are cut into segments according to ‘segment size’. First, SUSY computes cross-correlations within each segment across a certain range of lags. For example, for a parameter setting ‘maximum lag’ = ±3 s, all cross-correlations within a six-second window are considered. Twofold aggregation of these cross-correlations (across all segments and lags) yields a measure for real synchrony. Beforehand, cross-correlations are Fisher’s *Z*-transformed to allow for aggregation. SUSY provides two indices based on absolute and non-absolute *Z* values: ${\bar{Z}}_{\mathrm{noabs}}$ (SUSY) and ${\bar{Z}}_{\mathrm{abs}}$ (SUSY). Whereas ${\bar{Z}}_{\mathrm{abs}}$ (SUSY) indicates overall synchrony, ${\bar{Z}}_{\mathrm{noabs}}$ (SUSY) distinguishes between in-phase and anti-phase synchrony. The complete procedure of dyadic SUSY generates a surrogate control condition for ${\bar{Z}}_{\mathrm{noabs}}$ and ${\bar{Z}}_{\mathrm{abs}}$ by shuffling the sequence of segments of the original time series, so that segments of A are ‘falsely’ aligned with segments of B. Shuffling can be repeated and produces many different surrogates. Then ${\bar{Z}}_{\mathrm{noabs}-\mathrm{surr}}$ (SUSY) and ${\bar{Z}}_{\mathrm{abs}-\mathrm{surr}}$ (SUSY) as markers of surrogate synchrony are computed. Mathematical details of SUSY methodology were described by Tschacher and Haken [38] and Tschacher and Meier [6].

Surrogate Concordance (SUCO, cf. www.embodiment.ch, accessed on 11 October 2021) has its origins in the concordance approach by Marci and Orr [15] and was first implemented in Tschacher and Meier [6]. SUCO requires four basic parameters: ‘segment size’, ‘window size’, ‘lag’, and ‘increment’. A local slope is computed inside a window by least squares regression in the corresponding segments of A and B. Each window is shifted according to ‘increment’ until all windows per segment are taken into account. Synchrony is defined as correlation of A’s and B’s local slopes. Then, after Fisher’s *Z* transformation of correlations, these are aggregated across all segments of the time series. All procedures can be performed with lagged windows. SUCO yields two dyadic synchrony indices, ${\bar{Z}}_{\mathrm{abs}}\;$(SUCO) and ${\bar{Z}}_{\mathrm{noabs}}$ (SUCO) representing real synchrony. Again, surrogate analysis is performed by random shuffling of segments, defining the indices for surrogate synchronies.

### 2.4. Multivariate Surrogate Synchrony Computation

Multivariate Surrogate Synchrony (mv-SUSY) estimates the synchrony within datasets that contain more than two time series. The number of time series is denoted by *m*. mv-SUSY was adapted and extended from SUCO and SUSY with respect to implementing the surrogate controls [3,8]. As in dyadic synchrony, two computation steps are conducted to compare synchrony of the time series to surrogate synchrony of segment-shuffled surrogate time series. According to ‘segment size’, all *m* time series of the *m*-variate dataset are cut into equal-sized segments. The number of segments follows from the duration (number of observations divided by sampling rate) for each time series divided by segment size. For example, a dataset with *m* = 10 time series of 5 min (9000 observations at 30 Hz) and ‘segment size’ set to five seconds contains 60 segments. Each segment renders one synchrony value.

We developed two methods to assess mv-SUSY: omega and lambda_max_. omega is a measure of multivariate synchrony that makes use of the actually measured degree of entropy H_act_ (actual entropy). Entropy is a measure of disorder of a dataset in thermodynamics, with its equivalent Shannon information in information theory [39]. Landsberg [40] suggested to normalize entropy by the maximum entropy possible in a system H_pot_ (potential entropy), thus H_act_/H_pot_. As this ratio assumes values between 0 and 1, ‘Landsberg order’ consequently becomes omega = 1 − H_act_/H_pot_. Banerjee et al. [41] proposed to estimate these entropies based on the variance-covariance matrix, which avoids specific problems of assessing entropies in psychological and biological systems [42,43]. Thus, entropies are derived from the covariance matrix of a dataset. We used the absolute values of all covariances to indicate that also negative covariances contribute positively to the overall covariance of the dataset. The variance-covariance matrix is a *m* × *m* symmetrical matrix whose cells contain the covariance coefficients, measures of linear relationship, of each pair of variables, and whose main diagonal represents the univariate variances of each variable. H_act_ is estimated from the determinant of this matrix as a measure of generalized variance. The normalized entropy is therefore the actual entropy in relation to the maximum potential entropy of a system. Maximum entropy corresponds to a state of total independence between the elements of a system, where the covariance coefficients would be zero and the determinant becomes the product of the diagonal. Thus, maximum entropy H_pot_ is the product of all univariate variances. In mv-SUSY, omega is computed as 1 − (H_act_/H_pot_) in each segment of the dataset, and these values are aggregated across all segments.

The second mv-SUSY method, lambda_max_, is computed by eigendecomposition of the correlation matrix for each segment. The correlation matrix is a *m × m* matrix where *m* corresponds to the number of dimensions of a dataset, in the present case the number of time series. The correlation matrix contains the correlation coefficients between two variables (−1 ≤ *r* ≤ 1) in the upper and lower triangle. All diagonal elements are equal to 1, the correlation of each variable with itself. The *m × m* correlation matrix can be decomposed into *m* eigenvalues *λ*, and *m* corresponding eigenvectors *v*. For details on the calculation of eigenvalues and eigenvectors see for example Fischer [44]. Eigenvalues are associated with the variances of the variables on which the correlation matrix is based. Geometrically, eigenvectors are orthogonal vectors scaled by their corresponding eigenvalues, indicating the multidimensional dispersion of the data. In detail, the proportion of variance associated with a particular dimension is equal to the corresponding eigenvalue divided by the sum of all eigenvalues. The sum of the eigenvalues refers to the sum of the diagonal elements, which in the case of the correlation matrix is always *m* [45]. We propose that multivariate synchrony can be defined by one or a few large eigenvalues, when the remaining eigenvalues are small. In this case, there are only a few dominant dimensions that account for most of the variance in the data. Thus, lambda_max_ in mv-SUSY is computed by the proportion of the largest eigenvalue to the sum of all eigenvalues for each segment. Eigenvalues and eigenvectors are also the fundament of principle component analysis (PCA). PCA prioritizes key dimensions, so-called principal components, for the use of dimensionality reduction. In the field of self-organization, Tschacher and Grawe [46] previously used the variance explained by the first component as a measure of order in continuously rated therapy session reports.

Finally, the estimated multivariate synchronies of both omega and lambda_max_ are controlled for random or spuriously inflated synchrony using surrogate analysis. Time series segmentation formed the base of surrogate analysis (step two) in order to reduce potential effects of non-stationarity of time series data. In the surrogate step, mv-SUSY randomly shuffles the segments of all *m* time series independently of each other. Only those surrogates are allowed that do not include segments that matched in the original dataset. From *m* time series with *s* segments, *s!*/(*s − m)!* surrogates can be generated. The parameter ‘number of surrogates’ determines the number of surrogates randomly drawn from the pool of surrogates to limit processing time. Randomized shuffling in step two provides surrogate time series that share important characteristics with the measured time series segments such as the length of segments, their means and standard deviations.

Synchrony of surrogates is computed in the same way as real synchrony, using the methods omega or lambda_max_. Finally, to obtain global measures for synchrony, we compute effect sizes, ES (mv), of omega and lambda_max_ as the difference between the respective synchrony and mean surrogate synchrony standardized by the standard deviation of surrogate synchronies.

### 2.5. Statistical Analysis

To investigate the first goal, mv-SUSY was applied to simulated datasets 1 to 5. The segment size parameter was set to five seconds. Assuming a sampling rate of 30 Hz, each segment comprised 1500 observations. The number of surrogates was limited to 1000. Addressing the second goal, we estimated synchrony for the empirical datasets 6 to 8, using mv-SUSY. The same parameter settings as for the simulated data were adopted for datasets 6 and 7. To account for the lower sampling rate in dataset 8, segment size was set to six seconds, thus the number of observations per segment was kept stable across datasets. Again, we randomly chose 1000 surrogate segments as a control for real synchrony. For goal one and two, we tested the null hypotheses that there were no differences between synchrony of the original time series and synchrony of surrogates using Wilcoxon rank tests.

To explore the third goal of the present article, dyadic SUCO and dyadic SUSY were applied to all datasets. To account for the multivariate nature of the datasets, we computed synchronies of all dyadic combinations of the *m* time series of each dataset (for *m* = 10, this yields (10 × 9)/2 = 45 dyads). With regard to parameter settings, a segment size of 20 s (datasets 1 to 7) and 24 s (dataset 8) was chosen for both dyadic approaches. We set the number of surrogates to the maximum (240 surrogates). The lag parameter in dyadic SUSY was fixed at ±3 s across datasets. For SUCO, linear slopes were computed within a three-second window, and the increment was one second. To obtain global synchrony measures, absolute and non-absolute effect sizes were aggregated across all 45 dyads for both approaches yielding ES_abs_ (SUCO), ES_noabs_ (SUCO), ES_abs_ (SUSY), and ES_noabs_ (SUSY). The general formula for effect sizes is ES = (mean($\bar{Z}$) − mean($\bar{Z}$surr))/SD($\bar{Z}$surr). For each dataset, we performed one-sample *t*-tests against the null hypothesis that the respective aggregated effect sizes, ES_noabs_, were not different from zero. Paired *t*-tests of ${\bar{Z}}_{\mathrm{abs}}$ and ${\bar{Z}}_{\mathrm{abs}-\mathrm{surr}}$ were conducted to test whether synchrony was present based on absolute values. In the case of negative absolute effect sizes, additional paired *t*-tests were considered redundant because surrogate synchrony exceeded real synchrony if ES_abs_ < 0. Statistical analyses and plots were performed using the software environment R [47].

## 3. Results

### 3.1. Simulated Data

The simulated data in datasets 1 to 5 were analyzed using the synchrony algorithms mv-SUSY, SUSY, and SUCO. Results are presented in Table 1.

Dataset 1 comprised stationary random time series. Multivariate synchrony estimated by the standardized differences between real synchrony and surrogate synchrony, ES (mv), was close to zero for lambda_max_ and omega. This was further confirmed by Wilcoxon rank tests revealing non-significant differences between real synchrony and surrogate synchrony for both mv-SUSY methods. Analysis of aggregated dyadic synchrony with SUSY and SUCO required paired *t*-tests (absolute values) and one-sample *t*-tests (non-absolute values). Paired *t*-testing for ${\bar{Z}}_{\mathrm{abs}}$ and ${\bar{Z}}_{\mathrm{abs}-\mathrm{surr}}$ revealed that real synchrony did not significantly exceed surrogate synchrony for SUCO (*t*(44) = −1.04, ns). Negative ES_abs_ (SUSY) showed that surrogate synchrony was even higher compared to real synchrony. Thus, comparison of means by paired *t*-testing was redundant. Both non-absolute effect sizes, ES_noabs_ (SUSY) and ES_noabs_ (SUCO), did not differ from zero. Accordingly, mv-SUSY, SUSY, and SUCO did not indicate synchrony in dataset 1.

The same procedure was used to investigate non-stationary time series with linear trends (dataset 2). With regard to mv-SUSY, Wilcoxon rank tests revealed no significant differences between real synchrony and surrogate synchrony (lambda_max_ and omega). SUCO and SUSY confirmed these results for dyadic synchrony. Non-absolute effect sizes revealed absence of synchrony. This was found for synchrony based on window-wise slopes (SUCO) as well as for synchrony estimated by cross-correlations (SUSY). Results were the same for absolute values. The difference between real synchrony and surrogate synchrony was not significant in SUCO (*t*(44) = 1.51, ns) and SUSY (*t*(44) = 0.68, ns). Thus, the multivariate approaches as well as the dyadic approaches did not indicate presence of synchrony.

We evaluated autocorrelated time series in dataset 3. Results demonstrated no synchrony estimated by the mv-SUSY approach. In synchrony aggregated across dyads a less clear picture emerged. For SUCO, surrogate synchrony was significantly higher compared to real synchrony (*t*(44) = 2.34, *p* = 0.02). However, this was only the case for absolute values. ES_noabs_ (SUCO) did not indicate synchrony. SUSY showed absence of synchrony consistently. Absolute effect size, ES_abs_ (SUSY), was negative and non-absolute effect size, ES_noabs_ (SUSY), did not significantly differ from zero. Hence, we did not find synchrony for autocorrelated time series with one exception, namely SUCO (absolute values).

The SUCO, SUSY and mv-SUSY approaches were used to investigate the presence of synchrony in oscillatory time series (dataset 4). Both mv-SUSY methods, lambda_max_ and omega, revealed high values for real synchrony. Effect sizes remained small and non-significant due to high surrogate synchrony. SUCO and SUSY showed similar results. Non-absolute effect sizes did not differ from zero for both approaches. Negative absolute effect sizes in SUCO indicated that real synchrony was lower compared to surrogate synchrony. Furthermore, real synchrony did not differ from surrogate synchrony estimated by SUSY (*t*(44) = 0.56, ns). For dataset 4, mv-SUSY, SUSY and SUCO demonstrated no synchrony.

Analysis of dataset 5 addressed multivariate correlation among time series. Difference of real synchrony and surrogate synchrony was significant for lambda_max_ and omega indicated by high, positive effect sizes. The non-absolute effect size for SUCO did significantly differ from zero. In addition, the difference between real synchrony and surrogate synchrony estimated by SUCO was highly significant (*t*(44) = 43.80, *p* < 0.0001). Higher absolute effect sizes suggest that positive and negative correlations canceled each other out in the non-absolute effect sizes. Aggregated real synchrony exceeded surrogate synchrony in SUSY as well (*t*(44) = 47.52, *p* < 0.0001). The mean effect sizes across dyads differed significantly from zero (absolute values). Thus, all approaches demonstrated substantial synchrony for dataset 5.

In sum, hypothesis 1a (“no spurious synchrony detection with mv-SUSY in datasets 1 to 4”) was supported. Hypothesis 1b (“synchrony detection with mv-SUSY in correlated dataset 5”) was also supported. Hypothesis 3 (“validation of mv-SUSY by dyadic SUSY and SUCO”) was supported in four of the five datasets. In dataset 3 the absolute effect size values and further *t*-test indicated significant differences between real synchrony and surrogate synchrony (SUCO).

### 3.2. Empirical Data

In Table 2, we present findings of mv-SUSY, SUSY, and SUCO for datasets 6 to 8. Results of synchrony analysis in dataset 6 (Kinect intraindividual movement data) showed significant differences between real synchrony and the surrogate synchrony using the mv-SUSY approach. This was found for both lambda_max_ and omega. SUSY and SUCO suggested presence of high synchrony as well. The dyadic-based approaches revealed large and positive non-absolute effect sizes, which differed significantly from zero. Furthermore, paired *t*-tests revealed significant differences between real synchrony and surrogate synchrony for absolute values estimated by SUCO (*t*(44) = 8.23, *p* < 0.0001) as well as SUSY (*t*(44) = 14.65, *p* < 0.0001). Accordingly, presence of synchrony in intraindividual movement data (participant 1) was confirmed by mv-SUSY, SUSY and SUCO.

With regard to the second Kinect movement dataset, dataset 7, the same pattern emerged. mv-SUSY methods omega and lambda_max_ showed lower synchrony in surrogates. Wilcoxon rank tests revealed that this difference was significant. As in dataset 6, multivariate synchrony reached a maximum with aggregated synchrony = 1 for omega. Non-absolute effect sizes pointed toward high synchrony based on window-wise slopes (SUCO) as well as the windowed cross-correlation approach (SUSY). Paired *t*-tests for absolute values indicated synchrony in SUCO (*t*(44) = 8.80, *p* < 0.0001). So did the results for SUSY comparing real synchrony to surrogate synchrony (*t*(44) = 18.74, *p* < 0.0001). The findings for the intraindividual movement data (participant 2) consistently indicated multivariate synchrony across all approaches.

Synchrony measures mv-SUSY, SUSY and SUCO were applied to dataset 8 (movement coordination of participants of a group discussion). Findings indicated higher effect sizes for omega compared to lambda_max_. Nevertheless, both methods clearly showed presence of synchrony. Whereas the one-sample *t*-test revealed significant non-absolute effect sizes, the paired *t*-test of absolute values did not reach significance for SUCO (*t*(44) = 1.38, ns). SUSY results presented an unambiguous picture. Non-absolute effect sizes against zero as well as real synchrony compared to surrogate synchrony reached significance (*t*(44) = 2.14, *p* < 0.05).

Taken together, hypothesis 2 (“synchrony detection with mv-SUSY in empirical datasets 6 to 8”) was accepted. In 2 of 3 datasets we further confirmed hypothesis 3 (“validation of mv-SUSY by dyadic SUSY and SUCO”). In dataset 8 the absolute effect size values and further *t*-test showed that real synchrony did not significantly exceed surrogate synchrony (SUCO).

## 4. Discussion

The methodology of synchrony research should move beyond dyadic measures alone. Dyadic synchronies are of course well-justified as signatures of the relationship between therapist and client, or spouses of a couple, or the participants of conversation between two people. Yet, how may we address the synchronization of more complex phenomena such as groups, multi-person therapies, or generally dynamical systems that comprise more than just two variables? The present article therefore introduced a multivariate approach to quantify synchrony in more complex multidimensional systems. We presented the novel mv-SUSY methodology to estimate multivariate synchrony, and examined its functionality in eight datasets that exemplify different types of longitudinal patterns.

Our first goal was to investigate the performance of mv-SUSY in five simulated time series. Analysis of simulated time series in the context of synchrony has been used before. This was done to introduce new computational methods [48] or in surrogate analysis [36]. The time series in the current study comprised autocorrelation, trends, and seasonality which are common properties of empirical time series, for example in physiology (e.g., electrodermal activity, respiration, heart beats) and movement data. Results confirmed the hypothesized detection and rejections of synchrony. Both mv-SUSY methods, lambda_max_ and omega, revealed similar patterns of real and surrogate synchrony across datasets. We found that real synchrony (cf Table 1) was rather low in stationary random time series (dataset 1) and non-stationary time series with linear trends (dataset 2). In dataset 1, small real synchrony values resulted from intercorrelations close to zero among white noise data (see intercorrelations in the methods section). Dataset 2 time series correlated substantially because of their stable linear trends. Segment-wise computation of real synchrony allowed to account for these overarching trends. Thus, we found comparable degrees of real synchrony in white noise and non-stationary linear trends of otherwise unrelated time series. mv-SUSY did correctly reject synchrony for stationary random data as well as non-stationary linear trend data despite its temporal dependency. Autocorrelated (dataset 3) and oscillation time series (dataset 4) showed higher levels of real synchrony compared to the first two datasets. The lack of significance here resulted from similarly high levels of synchrony in the surrogates. This finding can be explained by the covariance respectively correlation-based nature of mv-SUSY. Autocorrelation and oscillation represent high dependency between observations at different time points thus provoking substantial segment-wise correlations. Surrogate testing controlled successfully for these reoccurring data properties, thus avoiding false-positive synchrony findings for uncorrelated time series. Not only the absence but also the presence of synchrony was found as assumed. In concordance with our hypothesis, mv-SUSY indicated a considerable degree of synchrony in multivariate correlated data (dataset 5).

We examined mv-SUSY for movement data in goal two. Synchrony was present in a dyadic body conversation task. This experimental task was designed to limit interactive dynamics to one single modality, dance-like movements, while allowing focusing on the whole body [37]. Both participants (dataset 6 and dataset 7) demonstrated significant coordination between their limbs, respectively, which was also found in the original study. There, Galbusera and colleagues [37] investigated interpersonal and intrapersonal synchrony and its association with self-regulation of emotion in 66 adults undergoing the body conversation task. In the present study, mv-SUSY further revealed synchrony in body movement (upper body region) in a virtual group discussion of 10 individuals. Accordingly, we found above-random coordination between the members of this discussion.

The third goal was to compare mv-SUSY performance with well-established dyadic synchrony approaches. SUSY and SUCO replicated mv-SUSY findings to a large extent. There were few cases of divergent results that did not support the hypotheses. First, SUCO indicated synchrony for dataset 3 (autocorrelated time series). However, this was only the case for absolute values. Since we did not find similar results in oscillation or trend data, which also showed a reasonable degree of autocorrelation, there is little evidence that SUCO is prone to autocorrelation. Second, SUCO did not find synchrony in dataset 8. Again, this was only the case for absolute values. As surrogate synchrony was lower compared to real synchrony, the difference pointed toward synchrony but it did not reach significance. Overall, analysis based on mv-SUSY, SUSY, and SUCO resulted in similar estimations of synchrony. Rather than competing with each other, each algorithm covers unique aspects with regard to synchrony analysis. One main feature of SUSY is the computation of cross-correlations. Accordingly, delayed responses in a predefined window are taken into account. SUSY and SUCO allow differentiating between in-phase and anti-phase synchrony. This is of considerable relevance in social contexts where each member has a specific role, e.g., in patient-therapist interaction.

Findings of the present study underline the feasibility of estimating synchrony within groups of individuals by mv-SUSY. Multi-person synchrony, although based on the aggregation of dyadic synchrony, has been investigated across disciplines. For example, researchers found physiological synchrony in audience members of classical concerts [25], brain-to-brain synchrony in a classroom [49], and physiological synchrony in teams of three during a cooperative production task [50]. Other studies investigated the effects of experimentally manipulated synchrony compared to control conditions. In a meta-analysis of 42 studies, synchrony in groups was associated with prosocial behavior, perceived social bonding, social cognition, and positive affect with effect sizes ranging from 0.11 to 0.28 [51]. Recently, multivariate synchrony approaches have been proposed, yet these have limitations such as the absence of surrogate controls that should be addressed. mv-SUSY complements the available methods by providing two novel synchrony measures (lambda_max_ and omega), which are applicable to oscillatory, non-oscillatory, and non-stationary time series, and are adequately controlled by surrogate analysis.

Analysis of synchrony corresponds to dynamical systems theory, which originated from mathematics and physics, but is increasingly used to focus on the change over time in biological, cognitive, or social systems [39,52]. At its core, the theory assumes that collective behavior may result from self-organization in interacting components of the respective system and thus exhibit processes of emergence. In line with these ideas, the study of synchrony accounts for the time-evolving pattern in individuals’ time series. For an overview regarding complex dynamical systems in social and personality psychology, see for example Richardson et al. [53]. Furthermore, the meaning of nonverbal synchrony is associated with the embodiment concept. The embodiment paradigm is a vibrant field that can be seen as the preliminary endpoint of the historical development from behaviorism to cognitivism to embodied cognition. According to the 4E paradigm, cognition is embodied, enactive, embedded and extended [54]. Taken together, to study social interaction between individuals, one has to consider dynamic processes, i.e., change over time. These may lead to coordinated, self-organized patterns of behavior, where individuals form a coupled emerging system which is said to be coordinated.

### 4.1. Strengths and Limitations

Several strengths and limitations of the study are noteworthy. The methodological approach was appropriate to the research questions and allowed (a) the application of mv-SUSY to simulated and empirical data, and (b) the comparison of the results with established dyadic approaches. mv-SUSY is based on general mathematical principles, namely order analysis and eigendecomposition. These concepts are of widespread use in statistical analysis, for example in principle component analysis, which is commonly used for dimensionality reduction in large datasets.

Further strengths and weaknesses of the study are closely linked to the differentiation of dyadic and multivariate synchrony analysis. Analysis of multivariate time series with dyadic synchrony approaches generates increased number of comparisons and further leads to a hierarchical data structure. Yet mv-SUSY provides one global measure of synchrony for multiple variables thus minimizing the number of aggregation steps. In future research implementations, mv-SUSY however still allows for estimating the degree of individual contributions to group synchrony, for instance by stepwise exclusion of the respective group member.

In line with other dyadic approaches such as SUSY and SUCO, mv-SUSY provides an appropriate control condition to determine above-random synchrony. According to a recent systematic review, “To validate findings, a null hypothesis determining the potential for chance findings of PS [physiological synchrony] in contextually matched, randomized data is often necessary. Otherwise, it may be unclear whether results are valid, or due to chance” [13]. Since surrogate analysis based on segment shuffling uses inherent characteristics of the respective data set, it is a conservative form of a surrogate synchrony control condition. In contrast to participant shuffling, the procedure in mv-SUSY, SUSY, and SUCO does not require additional data.

A limitation of the presented approach lies in the dependency on parameter settings. mv-SUSY adds group size to the current set of parameters. The effect of group size needs to be investigated in future studies.

### 4.2. Implications and Future Research

The present study emphasizes the importance of considering multiple variables in synchrony research, whether these variables are assessed within or across individuals. Recent technological advances allow researchers to record behavior or physiology of more than two individuals to obtain high-frequency multivariate time series. We introduced mv-SUSY as a computational approach to estimate synchrony based on such multivariate datasets. This complements current auto- or cross-correlational methods, which were limited to two variables.

The application to multivariate time series makes mv-SUSY a versatile methodology. mv-SUSY can be used to investigate nonverbal synchrony across modalities. Current research mainly addresses traditional measures such as movement behavior or physiology (e.g., cardiac activity) in synchrony analysis. Other modalities have not received that much attention yet, for example, there is limited but promising evidence on hormonal synchrony [55] or synchrony in eye gaze [56]. These measures represent potential starting points to quantify multivariate synchrony by mv-SUSY. Future studies should not only focus on different measures but should strive for an integration of synchrony across multiple levels. Such studies would help to gain deeper insights in the embodiment of social interaction.

Furthermore, mv-SUSY can be applied to a variety of research fields to expand our understanding of interpersonal coordination. Future research might focus on the role of multivariate synchrony in clinical psychology and psychotherapy. Nonverbal synchrony has been associated with so-called common factors that represent the underlying change mechanisms in psychotherapy, such as the working alliance [57]. Advancing empirical work in this area includes shifting the perspective from single patients to multi-person settings (e.g., couple therapy, support groups). This will have important practical implications for group-based therapies leading to improved mental health care. In general, future studies should not only go beyond dyads but also beyond disciplines. At the intersection of psychology, musicology, and cultural studies particularly fruitful options arise. mv-SUSY may be applied to explore the presence of synchrony in ecological valid settings (e.g., school, sports, concerts). For example, mv-SUSY can help identify synchrony in concert audiences enriching the exploration of aesthetic experiencing [25]. Experimental research designs may also be used to investigate the association between synchrony measured by mv-SUSY and work-team cooperation under different cooperative task conditions.

In summary, with the introduction of mv-SUSY, we have provided a valid method based on the theoretical framework of dynamical systems theory to estimate intrapersonal and interpersonal synchrony in complex data.

## Figures and Tables

**Table 1:** Synchrony results for simulated data (datasets 1 to 5).

Dataset 1: stationary random time series				
	**mv-SUSY**		**SUCO**	**SUSY**
method	*lambda_max_*	*omega*	window-wise slopes	cross-correlation
Hertz	30	30	30	30
number of surrogates	1000	1000	240	240
segment size [s]	5	5	20	20
window size [s]			3	
lag [s]				–3 ≤ lag ≤ 3
real synchrony	14.22	0.23	${\bar{Z}}_{\mathrm{abs}}$ = 0.23, ${\bar{Z}}_{\mathrm{noabs}}$ = 0.01	${\bar{Z}}_{\mathrm{abs}}$ = 0.03, ${\bar{Z}}_{\mathrm{noabs}}$ = 0.00
surrogate synchrony	14.26	0.23	${\bar{Z}}_{\mathrm{abs}-\mathrm{surr}}$ = 0.23, ${\bar{Z}}_{\mathrm{noabs}-\mathrm{surr}}$ = 0.00	${\bar{Z}}_{\mathrm{abs}-\mathrm{surr}}$ = 0.03, ${\bar{Z}}_{\mathrm{noabs}-\mathrm{surr}}\;$= 0.00
ES (mv)	−0.05, *W* = 33315.00 (ns)	−0.03, *W* = 33379.00 (ns)		
ES_noabs_ (SUCO)			0.02, *t* = 0.11 (ns)	
ES_abs_ (SUCO)			0.09	
ES_noabs_ (SUSY)				0.01, *t* = 0.19 (ns)
ES_abs_ (SUSY)				−0.03
Dataset 2: non-stationary time series with linear trends				
	**mv-SUSY**		**SUCO**	**SUSY**
method	*lambda_max_*	*omega*	window-wise slopes	cross-correlation
Hertz	30	30	30	30
number of surrogates	1000	1000	240	240
segment size [s]	5	5	20	20
window size [s]			3	
lag [s]				–3 ≤ lag ≤ 3
real synchrony	14.31	0.23	${\bar{Z}}_{\mathrm{abs}}$ = 0.24, ${\bar{Z}}_{\mathrm{noabs}}$ = 0.01	${\bar{Z}}_{\mathrm{abs}}$ = 0.04, ${\bar{Z}}_{\mathrm{noabs}}$ = 0.01
surrogate synchrony	14.26	0.23	${\bar{Z}}_{\mathrm{abs}-\mathrm{surr}}$ = 0.23, ${\bar{Z}}_{\mathrm{noabs}-\mathrm{surr}}$ = 0.00	${\bar{Z}}_{\mathrm{abs}-\mathrm{surr}}$ = 0.04, ${\bar{Z}}_{\mathrm{noabs}-\mathrm{surr}}$ = 0.01
ES (mv)	0.07, *W* = 31310.00 (ns)	0.03, *W* = 30965.00 (ns)		
ES_noabs_ (SUCO)			0.10, *t* = 0.55 (ns)	
ES_abs_ (SUCO)			0.17	
ES_noabs_ (SUSY)				0.05, *t* = 0.42 (ns)
ES_abs_ (SUSY)				−0.01
Dataset 3: lag 1 autocorrelated time series				
	**mv-SUSY**		**SUCO**	**SUSY**
method	*lambda_max_*	*omega*	window-wise slopes	cross-correlation
Hertz	30	30	30	30
number of surrogates	1000	1000	240	240
segment size [s]	5	5	20	20
window size [s]			3	
lag [s]				–3 ≤ lag ≤ 3
real synchrony	24.77	0.87	${\bar{Z}}_{\mathrm{abs}}$ = 0.27, ${\bar{Z}}_{\mathrm{noabs}}$ = 0.00	${\bar{Z}}_{\mathrm{abs}}$ = 0.09, ${\bar{Z}}_{\mathrm{noabs}}$ = 0.00
surrogate synchrony	24.84	0.88	${\bar{Z}}_{\mathrm{abs}-\mathrm{surr}}$ = 0.25, ${\bar{Z}}_{\mathrm{noabs}-\mathrm{surr}}$ = 0.01	${\bar{Z}}_{\mathrm{abs}-\mathrm{surr}}$ = 0.01, ${\bar{Z}}_{\mathrm{noabs}-\mathrm{surr}}$ = 0.00
ES (mv)	−0.02, *W* = 32714.00 (ns)	−0.14, *W* = 33537.00 (ns)		
ES_noabs_ (SUCO)			−0.06, *t* = −0.34 (ns)	
ES_abs_ (SUCO)			0.42	
ES_noabs_ (SUSY)				0.25, *t* = 0.98 (ns)
ES_abs_ (SUSY)				−0.10
Dataset 4: sine oscillations				
	**mv-SUSY**		**SUCO**	**SUSY**
method	*lambda_max_*	*omega*	window-wise slopes	cross-correlation
Hertz	30	30	30	30
number of surrogates	1000	1000	240	240
segment size [s]	5	5	20	20
window size [s]			3	
lag [s]				–3 ≤ lag ≤ 3
real synchrony	24.79	0.98	${\bar{Z}}_{\mathrm{abs}}$ = 0.22, ${\bar{Z}}_{\mathrm{noabs}}$ = 0.01	${\bar{Z}}_{\mathrm{abs}}$ = 0.04, ${\bar{Z}}_{\mathrm{noabs}}$ = 0.00
surrogate synchrony	24.83	0.98	${\bar{Z}}_{\mathrm{abs}-\mathrm{surr}}$ = 0.22, ${\bar{Z}}_{\mathrm{noabs}-\mathrm{surr}}$ = 0.00	${\bar{Z}}_{\mathrm{abs}-\mathrm{surr}}$ = 0.04, ${\bar{Z}}_{\mathrm{noabs}-\mathrm{surr}}$ = 0.00
ES (mv)	−0.01, *W* = 32178.00 (ns)	0.13, *W* = 31729.00 (ns)		
ES_noabs_ (SUCO)			−0.20, *t* = −0.19 (ns)	
ES_abs_ (SUCO)			−0.04	
ES_noabs_ (SUSY)				−0.02, *t* = −0.25 (ns)
ES_abs_ (SUSY)				0.00
Dataset 5: correlated random time series				
	**mv-SUSY**		**SUCO**	**SUSY**
method	*lambda_max_*	*omega*	window-wise slopes	cross-correlation
Hertz	30	30	30	30
number of surrogates	1000	1000	240	240
segment size [s]	5	5	20	20
window size [s]			3	
lag [s]				–3 ≤ lag ≤ 3
real synchrony	83.67	1.00	${\bar{Z}}_{\mathrm{abs}}$ = 1.19, ${\bar{Z}}_{\mathrm{noabs}}$ = 1.19	${\bar{Z}}_{\mathrm{abs}}$ = 0.04, ${\bar{Z}}_{\mathrm{noabs}}$ = 0.01
surrogate synchrony	14.33	0.24	${\bar{Z}}_{\mathrm{abs}-\mathrm{surr}}$ = 0.23, ${\bar{Z}}_{\mathrm{noabs}-\mathrm{surr}}$ = 0.04	${\bar{Z}}_{\mathrm{abs}-\mathrm{surr}}$ = 0.03, ${\bar{Z}}_{\mathrm{noabs}-\mathrm{surr}}$ = 0.00
ES (mv)	96.47, *W* = 0.00 ****	17.33, *W* = 0.00 ****		
ES_noabs_ (SUCO)			11.93, *t* = 38.19 ****	
ES_abs_ (SUCO)			23.78	
ES_noabs_ (SUSY)				2.06, *t* = 46.04 ****
ES_abs_ (SUSY)				4.12

Note. **** *p* < 0.0001; ns, non-significant.

**Table 2:** Synchrony results for empirical movement data (datasets 6 to 8).

Dataset 6: Kinect, coordinated intraindividual movement (participant 1)				
	**mv-SUSY**		**SUCO**	**SUSY**
method	*lambda_max_*	*omega*	window-wise slopes	cross-correlation
Hertz	30	30	30	30
number of surrogates	1000	1000	240	240
segment size [s]	5	5	20	20
window size [s]			3	
lag [s]				–3 ≤ lag ≤ 3
real synchrony	47.38	1.00	${\bar{Z}}_{\mathrm{abs}}$ = 0.92, ${\bar{Z}}_{\mathrm{noabs}}$ = 0.90	${\bar{Z}}_{\mathrm{abs}}$ = 0.12, ${\bar{Z}}_{\mathrm{noabs}}$ = 0.07
surrogate synchrony	21.01	0.69	${\bar{Z}}_{\mathrm{abs}-\mathrm{surr}}$ = 0.23, ${\bar{Z}}_{\mathrm{noabs}-\mathrm{surr}}$ = −0.01	${\bar{Z}}_{\mathrm{abs}-\mathrm{surr}}$ = 0.08, ${\bar{Z}}_{\mathrm{noabs}-\mathrm{surr}}$ = 0.00
ES (mv)	9.77, *W* = 51.00 ****	2.97, *W* = 0.00 ****		
ES_noabs_ (SUCO)			14.80, *t* = 10.63 ****	
ES_abs_ (SUCO)			12.29	
ES_noabs_ (SUSY)				13.02, *t* = 15.34 ****
ES_abs_ (SUSY)				8.35
Dataset 7: Kinect, coordinated intraindividual movement (participant 2)				
	**mv-SUSY**		**SUCO**	**SUSY**
method	*lambda_max_*	*omega*	window-wise slopes	cross-correlation
Hertz	30	30	30	30
number of surrogates	1000	1000	240	240
segment size [s]	5	5	20	20
window size [s]			3	
lag [s]				–3 ≤ lag ≤ 3
real synchrony	47.46	1.00	${\bar{Z}}_{\mathrm{abs}}$ = 0.89, ${\bar{Z}}_{\mathrm{noabs}}$ = 0.88	${\bar{Z}}_{\mathrm{abs}}$ = 0.13, ${\bar{Z}}_{\mathrm{noabs}}$ = 0.08
surrogate synchrony	21.28	0.69	${\bar{Z}}_{\mathrm{abs}-\mathrm{surr}}$ = 0.23, ${\bar{Z}}_{\mathrm{noabs}-\mathrm{surr}}$ = 0.00	${\bar{Z}}_{\mathrm{abs}-\mathrm{surr}}$ = 0.09, ${\bar{Z}}_{\mathrm{noabs}-\mathrm{surr}}$ = 0.00
ES (mv)	9.49, *W* = 0.00 ****	2.91, *W* = 0.00 ****		
ES_noabs_ (SUCO)			12.56, *t* = 10.86 ****	
ES_abs_ (SUCO)			15.15	
ES_noabs_ (SUSY)				18.40, *t* = 25.24 ****
ES_abs_ (SUSY)				11.69
Dataset 8: Zoom motion energy, group discussion				
	**mv-SUSY**		**SUCO**	**SUSY**
method	*lambda_max_*	*omega*	window-wise slopes	cross-correlation
Hertz	25	25	25	25
number of surrogates	1000	1000	240	240
segment size [s]	6	6	24	24
window size [s]			3	
lag [s]				–3 ≤ lag ≤ 3
real synchrony	16.14	0.36	${\bar{Z}}_{\mathrm{abs}}$ = 0.22, ${\bar{Z}}_{\mathrm{noabs}}$ = 0.04	${\bar{Z}}_{\mathrm{abs}}$ = 0.05, ${\bar{Z}}_{\mathrm{noabs}}$ = 0.01
surrogate synchrony	15.59	0.31	${\bar{Z}}_{\mathrm{abs}-\mathrm{surr}}$ = 0.21, ${\bar{Z}}_{\mathrm{noabs}-\mathrm{surr}}$ = 0.01	${\bar{Z}}_{\mathrm{abs}-\mathrm{surr}}$ = 0.04, ${\bar{Z}}_{\mathrm{noabs}-\mathrm{surr}}$ = 0.00
ES (mv)	0.39, *W* = 25525.00 **	0.51, *W* = 22495.00 ****		
ES_noabs_ (SUCO)			0.52, *t* = 2.82 **	
ES_abs_ (SUCO)			0.25	
ES_noabs_ (SUSY)				1.86, *t* = 3.69 ***
ES_abs_ (SUSY)				0.58

Note. ** *p* < 0.01; *** *p* < 0.001; **** *p* < 0.0001.

## Data Availability

The simulated datasets generated and analyzed during the current study are available from the corresponding author upon reasonable request. The empirical datasets 6 and 7 are not publicly available, for contact see [37].

## References

[1] Varela F.J., Varela F.J., Shear J. (1999). Present-Time Consciousness. The View from within: First-Person Approaches to the Study of Consciosness.

[2] Fujiwara K., Daibo I. (2018). Affect as an Antecedent of Synchrony: A Spectrum Analysis with Wavelet Transform. Q. J. Exp. Psychol..

[3] Tschacher W., Rees G.M., Ramseyer F. (2014). Nonverbal Synchrony and Affect in Dyadic Interactions. Front. Psychol..

[4] Lindenberger U., Li S.-C., Gruber W., Müller V. (2009). Brains Swinging in Concert: Cortical Phase Synchronization While Playing Guitar. BMC Neurosci..

[5] Kleinbub J.R. (2017). State of the Art of Interpersonal Physiology in Psychotherapy: A Systematic Review. Front. Psychol..

[6] Tschacher W., Meier D. (2020). Physiological Synchrony in Psychotherapy Sessions. Psychother. Res..

[7] Ramseyer F. (2020). Motion Energy Analysis (MEA): A Primer on the Assessment of Motion from Video. J. Couns. Psychol..

[8] Ramseyer F., Tschacher W. (2011). Nonverbal Synchrony in Psychotherapy: Coordinated Body Movement Reflects Relationship Quality and Outcome. J. Consult. Clin. Psychol..

[9] Altmann U., Schoenherr D., Paulick J., Deisenhofer A.-K., Schwartz B., Rubel J.A., Stangier U., Lutz W., Strauss B. (2019). Associations between Movement Synchrony and Outcome in Patients with Social Anxiety Disorder: Evidence for Treatment Specific Effects. Psychother. Res..

[10] Paulick J., Deisenhofer A.-K., Ramseyer F., Tschacher W., Boyle K., Rubel J., Lutz W. (2018). Nonverbal Synchrony: A New Approach to Better Understand Psychotherapeutic Processes and Drop-Out. J. Psychother. Integr..

[11] Chartrand T.L., Lakin J.L. (2013). The Antecedents and Consequences of Human Behavioral Mimicry. Annu. Rev. Psychol..

[12] Imel Z.E., Barco J.S., Brown H.J., Baucom B.R., Baer J.S., Kircher J.C., Atkins D.C. (2014). The Association of Therapist Empathy and Synchrony in Vocally Encoded Arousal. J. Couns. Psychol..

[13] Palumbo R.V., Marraccini M.E., Weyandt L.L., Wilder-Smith O., McGee H.A., Liu S., Goodwin M.S. (2017). Interpersonal Autonomic Physiology: A Systematic Review of the Literature. Personal. Soc. Psychol. Rev..

[14] Di Mascio A., Boyd R.W., Greenblatt M., Solomon H.C. (1955). The Psychiatric Interview: A Sociophysiologic Study. Dis. Nerv. Syst..

[15] Marci C.D., Orr S.P. (2006). The Effect of Emotional Distance on Psychophysiologic Concordance and Perceived Empathy Between Patient and Interviewer. Appl. Psychophysiol. Biofeedback.

[16] Karvonen A., Kykyri V.-L., Kaartinen J., Penttonen M., Seikkula J. (2016). Sympathetic Nervous System Synchrony in Couple Therapy. J. Marital Fam. Ther..

[17] Chartrand T.L., Bargh J.A. (1999). The Chameleon Effect: The Perception–Behavior Link and Social Interaction. J. Pers. Soc. Psychol..

[18] Wiltermuth S.S., Heath C. (2009). Synchrony and Cooperation. Psychol. Sci..

[19] Guastello S.J., Mirabito L., Peressini A.F. (2020). Autonomic Synchronization under Three Task Conditions and Its Impact on Team Performance. Nonlinear Dyn. Psychol. Life Sci..

[20] Coutinho J., Oliveira-Silva P., Fernandes E., Gonçalves O.F., Correia D., Perrone Mc-Govern K., Tschacher W. (2019). Psychophysiological Synchrony During Verbal Interaction in Romantic Relationships. Fam. Process.

[21] Coutinho J., Pereira A., Oliveira-Silva P., Meier D., Lourenço V., Tschacher W. (2020). When Our Hearts Beat Together: Cardiac Synchrony as an Entry Point to Understand Dyadic Co-regulation in Couples. Psychophysiology.

[22] Azhari A., Lim M., Bizzego A., Gabrieli G., Bornstein M.H., Esposito G. (2020). Physical Presence of Spouse Enhances Brain-to-Brain Synchrony in Co-Parenting Couples. Sci. Rep..

[23] Feldman R., Magori-Cohen R., Galili G., Singer M., Louzoun Y. (2011). Mother and Infant Coordinate Heart Rhythms through Episodes of Interaction Synchrony. Infant Behav. Dev..

[24] Seibert C., Greb F., Tschacher W. (2019). Nonverbale Synchronie und Musik-Erleben im klassischen Konzert. Jahrb. Musikpsychol..

[25] Tschacher W., Greenwood S., Wald-Fuhrmann M., Czepiel A., Tröndle M., Meier D. (2021). Physiological Synchrony in Audiences of Live Concerts. Psychol. Aesthet. Creat. Arts.

[26] Bachrach A., Fontbonne Y., Joufflineau C., Ulloa J.L. (2015). Audience Entrainment during Live Contemporary Dance Performance: Physiological and Cognitive Measures. Front. Hum. Neurosci..

[27] Coco M.I., Dale R. (2014). Cross-Recurrence Quantification Analysis of Categorical and Continuous Time Series: An R Package. Front. Psychol..

[28] Frank T.D., Richardson M.J. (2010). On a Test Statistic for the Kuramoto Order Parameter of Synchronization: An Illustration for Group Synchronization during Rocking Chairs. Phys. Nonlinear Phenom..

[29] Richardson M.J., Garcia R.L., Frank T.D., Gergor M., Marsh K.L. (2012). Measuring Group Synchrony: A Cluster-Phase Method for Analyzing Multivariate Movement Time-Series. Front. Physiol..

[30] Wallot S. (2019). Multidimensional Cross-Recurrence Quantification Analysis (MdCRQA)—A Method for Quantifying Correlation between Multivariate Time-Series. Multivar. Behav. Res..

[31] Guastello S.J., Peressini A.F. (2017). Development of a Synchronization Coefficient for Biosocial Interactions in Groups and Teams. Small Group Res..

[32] Behrens F., Moulder R.G., Boker S.M., Kret M.E. (2020). Quantifying Physiological Synchrony through Windowed Cross-Correlation Analysis: Statistical and Theoretical Considerations. BioRxiv Prepr..

[33] Kleinbub J.R., Talia A., Palmieri A. (2020). Physiological Synchronization in the Clinical Process: A Research Primer. J. Couns. Psychol..

[34] Schoenherr D., Paulick J., Strauss B.M., Deisenhofer A.-K., Schwartz B., Rubel J.A., Lutz W., Stangier U., Altmann U. (2019). Identification of Movement Synchrony: Validation of Windowed Cross-Lagged Correlation and -Regression with Peak-Picking Algorithm. PLoS ONE.

[35] Strang A.J., Funke G.J., Russell S.M., Dukes A.W., Middendorf M.S. (2014). Physio-Behavioral Coupling in a Cooperative Team Task: Contributors and Relations. J. Exp. Psychol. Hum. Percept. Perform..

[36] Moulder R.G., Boker S.M., Ramseyer F., Tschacher W. (2018). Determining Synchrony between Behavioral Time Series: An Application of Surrogate Data Generation for Establishing Falsifiable Null-Hypotheses. Psychol. Methods.

[37] Galbusera L., Finn M.T.M., Tschacher W., Kyselo M. (2019). Interpersonal Synchrony Feels Good but Impedes Self-Regulation of Affect. Sci. Rep..

[38] Tschacher W., Haken H. (2019). The Process of Psychotherapy: Causation and Chance.

[39] Haken H., Portugali J. (2016). Information and Selforganization: A Unifying Approach and Applications. Entropy.

[40] Landsberg P.T. (1984). Can Entropy and “Order” Increase Together?. Phys. Lett. A.

[41] Banerjee S., Sibbald P.R., Maze J. (1990). Quantifying the Dynamics of Order and Organization in Biological Systems. J. Theor. Biol..

[42] Shiner J.S., Davison M., Landsberg P.T., Tschacher W., Dauwalder J.-P. (1999). On measures for order and its relation to complexity. Dynamics, Synergetics, Autonomous Agents.

[43] Tschacher W., Scheier C., Grawe K. (1998). Order and Pattern Formation in Psychotherapy. Nonlinear Dyn. Psychol. Life Sci..

[44] Fischer G. (2003). Lineare Algebra. Vieweg Studium.

[45] Hadd A.R., Rodgers J.L. (2020). Understanding Correlation Matrices.

[46] Tschacher W., Grawe K. (1996). Selbstorganisation in Therapieprozessen. Z. Für Klin. Psychol..

[47] (2020). R Core Team R: A Language and Environment for Statistical Computing.

[48] Liu S., Zhou Y., Palumbo R., Wang J.-L. (2016). Dynamical Correlation: A New Method for Quantifying Synchrony with Multivariate Intensive Longitudinal Data. Psychol. Methods.

[49] Dikker S., Wan L., Davidesco I., Kaggen L., Oostrik M., McClintock J., Rowland J., Michalareas G., Van Bavel J.J., Ding M. (2017). Brain-to-Brain Synchrony Tracks Real-World Dynamic Group Interactions in the Classroom. Curr. Biol..

[50] Mønster D., Håkonsson D.D., Eskildsen J.K., Wallot S. (2016). Physiological Evidence of Interpersonal Dynamics in a Cooperative Production Task. Physiol. Behav..

[51] Mogan R., Fischer R., Bulbulia J.A. (2017). To Be in Synchrony or Not? A Meta-Analysis of Synchrony’s Effects on Behavior, Perception, Cognition and Affect. J. Exp. Soc. Psychol..

[52] Haken H., Portugali J. (2021). Information and Self-Organization II: Steady State and Phase Transition. Entropy.

[53] Richardson K., Hart W., Breeden C.J., Tortoriello G.K., Kinrade C. (2020). Validating Circumplex Scales of Perceived Impairments and Benefits from Prototypically Problematic Interpersonal Tendencies. Psychol. Assess..

[54] Newen A., De Bruin L., Gallagher S. (2018). The Oxford Handbook of 4E Cognition.

[55] Liu S., Rovine M.J., Cousino Klein L., Almeida D.M. (2013). Synchrony of Diurnal Cortisol Pattern in Couples. J. Fam. Psychol..

[56] Tschacher W., Tschacher N., Stukenbrock A. (2021). Eye Synchrony: A Method to Capture Mutual and Joint Attention in Social Eye Movements. Nonlinear Dyn. Psychol. Life Sci..

[57] Koole S.L., Atzil-Slonim D., Butler E., Dikker S., Tschacher W., Forgas J.P., Crano W.D., Fiedler K. (2020). In sync with your shrink. Applications of Social Psychology.

